# Meckel’s Diverticulum Causing Ileal Volvulus and Peritonitis after a Recent Appendectomy: A Case Report and Literature Review—We Should Likely Resect an Incidental MD

**DOI:** 10.3390/life13101996

**Published:** 2023-09-30

**Authors:** Matteo Zanchetta, Davide Inversini, Vincenzo Pappalardo, Niccolo Grappolini, Marika Morabito, Simone Gianazza, Giulio Carcano, Giuseppe Ietto

**Affiliations:** 1 General, Emergency and Transplant Surgery Department, ASST Settelaghi, University of Insubria, 21100 Varese, Italymarika.morabito@gmail.com (M.M.);; 2 Department of Medicine and Innovation Technology, University of Insubria, 21100 Varese, Italy; 3 Department of Surgery, Cittiglio-Angera Hospital-ASST Settelaghi, 21100 Varese, Italy

**Keywords:** Meckel’s diverticulum, laparoscopy, peritonitis, emergency surgery, volvulus, appendectomy

## Abstract

Meckel’s diverticulum (MD) is the most common congenital anomaly of the gastrointestinal tract with a 1–3% prevalence in the general population. The surgical management of symptomatic MD is well described in the literature, but there is still no consensus on the indication for prophylactic resection of incidental asymptomatic MD. To address this issue, we extensively reviewed the current literature and report our experience with laparoscopic management of an unusual case of MD causing ileal volvulus and acute peritonitis two weeks after a laparoscopic appendectomy for acute gangrenous appendicitis performed in another hospital. A 50-year-old man presented to the emergency department with acute and severe abdominal pain, vomiting, and constipation. He had undergone a laparoscopic appendectomy for acute appendicitis two weeks before in another hospital. The patient was apyretic, distressed, and seeking an antalgic position. The abdomen was mildly distended and tender, and the Blumberg sign was mildly positive in the central quadrants. The clinical picture deteriorated with fever, peritonismus, and leukocytosis. A CT scan showed an ileo–ileal adhesion near the ileocolic junction and dilatation of the upstream loops with the air–fluid levels. Through an urgent laparoscopy, a necrotic mass, the MD, was wedge-resected, and the surrounding ileal volvulus derotated. The postoperative course was uneventful. There is no definitive consensus on the appropriate management of incidental asymptomatic MD, although several studies have attempted to identify guiding criteria. Features of the MD, the patient’s risk factors, clinical presentation, and surgical approach need to be considered to establish definitive guidelines for the management of incidental asymptomatic MD. In the absence of definitive guidelines, personal expertise and judgement are the main resources for the surgeon approaching an incidental asymptomatic MD.

## 1. Introduction

Meckel’s diverticulum (MD) is the most common congenital anomaly of the gastrointestinal (GI) tract, with a prevalence of 1–3% in the general population [1,2,3,4,5]. It is the result of incomplete obliteration of the vitelline (omphalomesenteric) duct, which connects the primitive intestine to the yolk sac in early fetal life [2,6]. By definition, MD is a true diverticulum, as it is surrounded by all the layers of the small bowel wall [7]. The right vitelline artery, which arises from the superior mesenteric artery, supplies the MD [8]. When present, the MD is located along the antimesenteric margin of the ileum at a mean distance of 52.4 cm (range, 7–100 cm) from the ileocecal valve (ICV) and has a mean length and diameter of 3 cm and 1.58 cm, respectively [1]. In about half of the cases, MD contains heterotopic mucosa within its wall: most commonly gastric mucosa (60%), followed by pancreatic acinar tissue (16%), and the remainder Brunner’s glands, pancreatic islets, colonic mucosa, hepatobiliary tissue, or a combination of these [3,9]. The presence of ectopic tissue within, especially the gastric type and in patients of younger age, tends to increase the risk of clinical manifestations from MD, mostly in the form of bleeding [1,10,11]. According to the literature, MD becomes symptomatic in 4–71% of cases, predominantly in the first three to four decades of life, and up to four times more frequently in men [1,12,13]. Symptomatic adult patients usually present with intestinal obstruction (35.6%), inflammation (29.4%), or intestinal bleeding (27.3%) [1]. In paediatric symptomatic patients, these clinical manifestations occur in 46.7%, 19.5%, and 25.3% of cases, respectively [1]. Elderly patients most commonly develop neoplastic degeneration within the MD tissue [1,14,15], often with a poor prognosis [16]. Complications of MD are hemorrhage, intestinal obstruction, volvulus, inflammation, and perforation [14]. Perforation or inflammation of the MD is occasionally misdiagnosed as acute appendicitis [1,17]. The mortality rate of MD is around 0.1% [18]. We report our experience of laparoscopic management of an unusual case of MD causing ileal volvulus and acute peritonitis only 15 days after laparoscopic appendectomy for acute appendicitis. We inquired if the diverticulum was intentionally left in place during the previous abdominal surgery. It is unclear if any effort was made to locate it during the procedure. Accordingly, assuming that it was deliberately left in place, we pondered whether the decision was appropriate or erroneous. To address this issue, a thorough literature review was conducted. The literature widely describes the surgical management of diverticulum, but there is no definitive consensus regarding its prophylactic resection. 

## 2. Case Presentation

**Figure 1:**
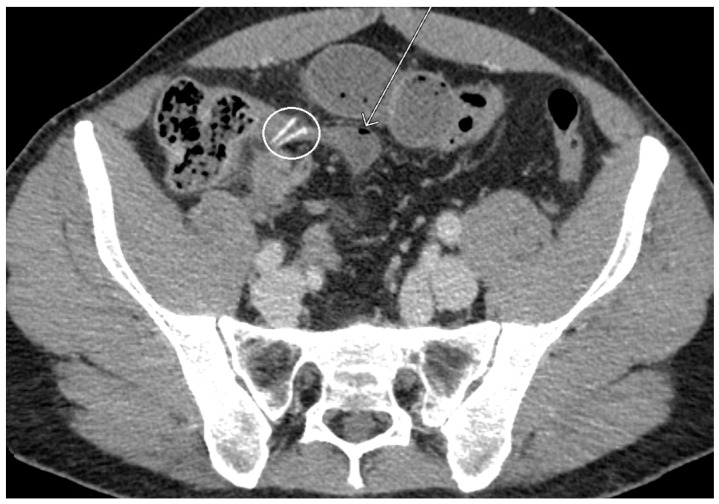
CT scan: suturing clips of previous appendectomy (circle); MD (arrow) whose pedunculated base can be seen in distal slices (Figure 2).

**Figure 2:**
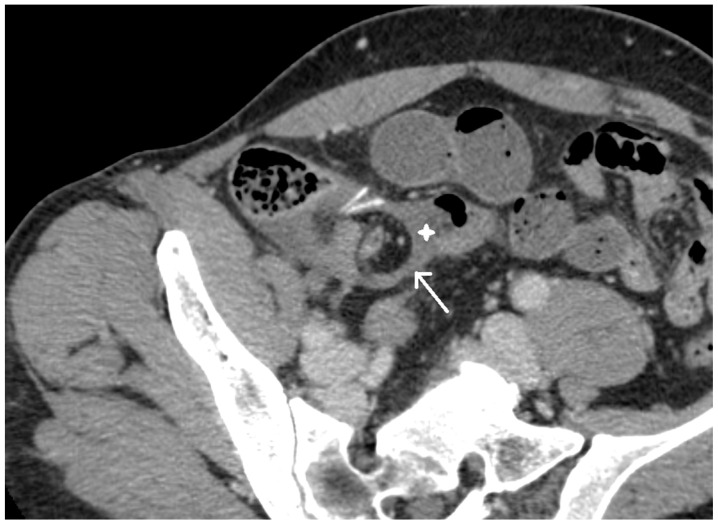
CT scan: pedunculated base of the MD (arrow) and MD (white star).

A 50-year-old man presented to the emergency department (ED) of our hospital (Ospedale di Circolo e Fondazione Macchi, Varese, Italy) with acute cramping and diffuse and severe (Numeric Pain Rating Scale 8) abdominal pain, associated with vomiting and constipation. He had undergone a laparoscopic appendectomy (LAE) for acute appendicitis two weeks before in another hospital. His past medical history includes an ischaemic stroke and a patent foramen ovale, currently treated with low-dose aspirin. The patient was apyretic, had a distressed and anxious appearance, and was seeking an antalgic position. On physical examination, scars from a previous surgery were physiologically healed; peristalsis was present on auscultation; the abdomen was mildly distended, tender all over, and the Blumberg sign was mildly positive in the central quadrants. The patient reported the passage of gas, but there was no fecal material on digital rectal examination. Initial laboratory tests were within normal limits (leukocytes 6.3 × 10^9^ units/L, PCR 1.0 mg/dL), except for a slight elevation in transaminases. A plain thoraco-abdominal x-ray showed a dilated stomach, coprostasis, no free air in the abdomen, and metallic clips from previous surgery. A computed tomography (CT) scan showed adhesion between two distal ileal loops near the ileocolic junction, thickening of the surrounding peritoneum, dilatation of the upstream intestinal loops with internal air–fluid levels, and no free fluid in the abdomen [Figure 1 and Figure 2]. 

**Figure 3:**
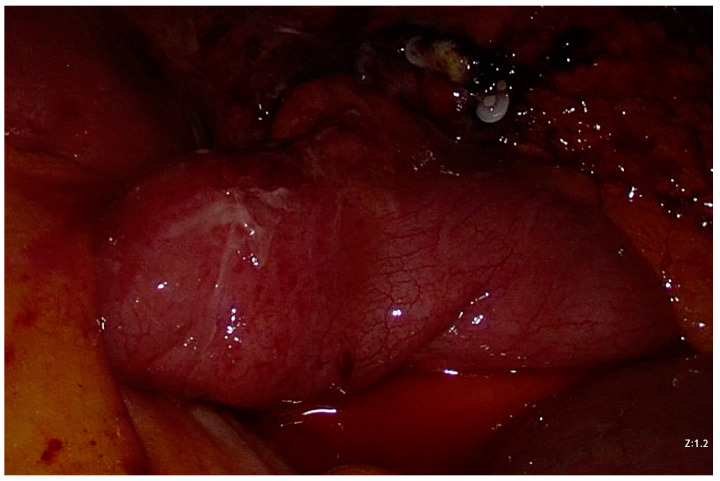
Intraoperative image: ileal volvulus, serosanguineous fluid, and clips from previous laparoscopic appendectomy. After derotating the volvulus, the necrotic mass (Figure 4) was more evident.

**Figure 4:**
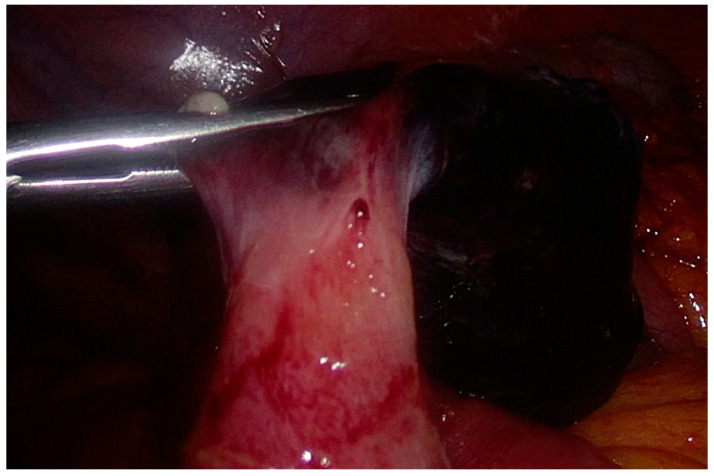
Intraoperative image: adjusting the mechanical stapler on the MD base (necrotic mass).

Initial conservative management (analgesic, antispasmodic, antiemetic, and gastroprotective drugs with parenteral hydration) failed, as the clinical condition rapidly deteriorated. The patient developed a fever, and his abdominal pain worsened with onset of diffuse peritonismus. Blood tests showed significant leukocytosis (12.53 × 10^9^ units/L). An urgent abdominal ultrasound (US) showed free fluid in the lower abdominal quadrants. An urgent laparoscopy was performed. The trocars were placed in the same positions as the previous LAE. On exploration, there was abundant serosanguinous fluid in the abdominal cavity [Figure 3]. Approximately 30 cm proximal to the ileocolic junction, there was an ileal volvulus around a necro-hemorrhagic mass originating from the ileal wall [Figure 3]. The surgeon derotated the volvulus. Then, the surgeon resected the necro-hemorrhagic mass with a mechanical stapler [Figure 4]. On anatomopathological examination, the mass was identified as an MD measuring 3 × 2.5 × 2 cm, with ischemic and infarcted areas and no evidence of heterotopic tissue. No neoplastic cells were found in an intraperitoneal fluid sample. The postoperative course was uneventful, and the patient was discharged on postoperative day (POD) 7. Follow-up was unremarkable.

## 3. Materials and Methods

The search source employed was the United States National Library of Medicine PubMed database. A comprehensive examination of past and recent publications was conducted using the “Incidental Meckel Diverticulum” search string. Publications were screened for their relevance, and selected articles were read in full. Their references were further scrutinized. Publications whose abstract contained the introduction, methods, results, and conclusion were included even when the full article was not extractable. The search was concluded in April 2023. 

## 4. Results

To obtain an insight into MD and conduct a thorough review of relevant literature on its management when incidentally found, a total of 58 publications, including case series, case reports, and reviews from 1962 to 2023, have been selected, analyzed, and reported. Six publications did not recommend the resection of an incidental MD [Table 1]. Thirty publications recommend the resection of an incidental MD whenever found or according to the presence of risk factors or other conditions [Table 2].

## 5. Discussion

Meckel’s diverticulum is a diagnostic challenge for clinicians because of its non-specific clinical manifestations. Meckel’s diverticulum is frequently discovered incidentally or during work-up for clinical conditions initially attributed to another cause (e.g., during videolaparoscopy). According to the literature, surgical resection, whether open or laparoscopic, is the treatment of choice for a symptomatic MD, when appropriate [1,50]. The extent of resection should be guided by the clinical presentation and intraoperative findings, with options ranging from a stapled diverticulectomy, or a limited wedge resection with closure of the ileal defect [51], to a more extensive bowel resection with subsequent loop anastomosis [52]. Meckel’s diverticulectomy has been demonstrated to be a safe method in the management of symptomatic MD in both adult and paediatric cohorts [50,53]. Morbidity was found to be higher after bowel resection than after simple diverticulectomy [49,54]. However, some advocate segmental bowel resection followed by anastomosis to ensure that no ectopic mucosa is left behind [43]. Taking a middle ground, Brungardt et al. in 2021 stated that diverticulectomy and small bowel resection are both likely to be appropriate approaches to the management of symptomatic MD when used in conjunction with a surgeon’s discretion, as there is no difference in outcomes between the two techniques [55]. Laparoscopic diverticulectomy is technically safe, cost-effective, and efficient in both emergency and elective settings [51,53,56], avoiding the morbidity associated with both open surgery and small bowel resection, with the added benefits of precise operative diagnosis and shorter recovery time [49,57]. 

However, despite decades of research, there is no consensus on the management and indication for possible prophylactic resection of incidental asymptomatic MD [58]. Most of the evidence related to MD comes from retrospective studies. There are only a few randomized studies, mostly single center, on complications, and none on incidental MD.

Early studies about asymptomatic MD were not in favor of prophylactic surgical resection. In 1962, Weinstein et al. reviewed 560 incidental MDs, of which 158 were left in situ on the basis that wide-mouthed diverticula (average width and length 1.92 cm × 2.99 cm, respectively) were not considered dangerous, and therefore their resection would only add to the risk of surgical complications and not provide any benefit [19]. In 1976, Soltero et al. estimated the lifetime risk of complications from MD to be 4.2%, eventually falling to 0% in old age; from then-published data, they calculated that it would be necessary to resect approximately 800 asymptomatic MDs to save one patient’s life [20]. In 1986, considering that the calculated lifetime risk of complications from MD is very low and that the risk of complications after diverticulectomy is not negligible, Leijonmarck et al. concluded that an incidental asymptomatic MD in adults should be left in situ [12]. In addition, they reported 28 cases of MD left in situ with no further clinical manifestations after an average of almost 8 years of follow up [12]. In 1993, DiGiacomo et al. stated that any incidental MD whose resection with a stapler would be unsafe and difficult because it is wide and short should be left in situ because of the low risk of complications [26]. In 1995, Peoples et al. stated that incidental diverticulectomy in adults should be abandoned, because the lifetime risk of developing complications does not significantly outweigh the surgical morbidity and mortality of resection [21].

In 1994, Cullen et al. concluded that incidental MDs should be removed to avoid future potential complications unless an additional condition (e.g., generalized peritonitis) would make resection hazardous [27]. In 1995, Matsagas et al. studied a population of 2074 patients undergoing AE with intraoperative evaluation for MD; they concluded that resection of the unexpected MD can be performed safely with a low complication rate, regardless of the patient’s age [28]. 

In 2004, Bani-Hani et al. stated that resection of incidentally found MDs does not increase operative morbidity and mortality and was therefore worthwhile, especially in young patients with long and narrow (diameter ≤ 2 cm) MDs [32]. Since then, many authors have advocated prophylactic resection of asymptomatic MDs [11,29,37,38,39,40,41,42,45,46,47] also, but not exclusively, to prevent potential future neoplastic degeneration [38,39,40,41,45,46]. A recent multicenter retrospective study by Tree et al. supports laparoscopic stapled resection of incidental asymptomatic MDs, considering the overall low complication rate and the potential for malignant transformation [49]. 

On the other hand, some other authors in the 2000s still prefer to leave an asymptomatic MD in situ. According to a 2004 observational study of 47 patients by Stone et al., removal of any asymptomatic MD should not be supported because complications of such a procedure, although uncommon, are often life-threatening [22]. In a 2008 review of the literature, Zani et al. did not support prophylactic resection of an incidental asymptomatic MD, noting that MD resection carried a 5.3% morbidity rate, mostly from wound infections, while leaving MDs in situ had an overall lower morbidity rate and lower long-term risk of complications [18]. 

A risk-based approach was first proposed in 1983 by Mackey et al.: they reviewed over 400 symptomatic MDs and identified some risk factors for complications such as age ≤ 40 years, length of the diverticulum ≥ 2 cm, presence of heterotopic tissue, and male sex [23]. In 2005, Park et al., after reviewing 1476 patients found to have an MD during surgery from 1950 to 2002, could neither definitively support nor reject the recommendation that every incidental asymptomatic MD should be removed but recommended removal of any incidental MD that had any of the four features (patient age younger than 50 years, male sex, diverticulum length greater than 2 cm, and the presence of histologically abnormal tissue) most commonly associated with clinical manifestations [14]. In the same year, after reviewing 233 cases of MD incidentally discovered during an appendectomy, Ueberrueck et al. stated that in cases of gangrenous or perforated appendicitis, an incidentally discovered MD should be left in place, whereas in an only mildly inflamed appendix it should be removed [33]. In 2006, Robijn et al. proposed a scoring system based on four risk factors (male sex, age < 45 years, diverticula > 2 cm, and the presence of a fibrous band) to guide the decision for surgery in incidental asymptomatic MDs [35]. This risk-based approach has been supported by a growing number of authors [24,25,30,31,34,36,43,44,48]. 

A recent systematic review has highlighted the contentious and unresolved nature of prophylactic resection concerning incidental MDs, indicating a tailor-made approach on a case-by-case basis as a potential solution [1]. In paediatric patients, incidental MD should be excised. In adult patients, incidental MD should also be removed based on the presence of risk factors (e.g., length greater than 2 cm) that may lead to complications. For elderly patients, incidental MD should be left in situ. Nevertheless, in all cases, it is crucial to keep the clinical picture in mind, as a silent MD may not take priority over life-threatening issues.

According to Mackey [23], Matsagas [28], Park [14], Robijn [35], and other more recent authors, some characteristics of the patient and the MD were clear indications to perform a prophylactic diverticulectomy. According to DiGiacomo, only the intraoperatively tested fit of the MD within the mechanical stapler could lead to a decision [26]. Considering the work of Hansen, although the size of the MD was an indication for prophylactic resection due to the risk of future complications, the severe intercurrent gangrenous appendicitis validly discouraged such an adjunctive surgical act [1]. According to Leijonmack, the size of our reported MD was not an indication for prophylactic diverticulectomy [12]. According to the conclusions of both Cullen [27] and Ueberrueck [33], gangrenous appendicitis with peritonitis was a contraindication for incidental removal of the MD, as the surgical act would have been riskier than leaving it in situ. 

Looking a posteriori at our reported case, not removing the MD during the previous LAE seems to be a mistake, but an a priori decision regarding its resection at such a time would have been controversial and could only have been based on the judgement, expertise, and competence of the treating surgeon. Therefore, although there is no definitive consensus regarding its prophylactic resection, and personal expertise remains the main resource for the surgeon, we could personally note a general trend in literature favoring the prophylactic resection. 

## 6. Conclusions

In conclusion, MD occurs in a minority of the population, and its clinical manifestations are largely non-specific. We managed life-threatening small bowel volvulus and peritonitis in a patient who had undergone LAE two weeks earlier, with the MD left in situ. There is no definitive consensus on the appropriate management of incidental asymptomatic MD. Over the decades, several studies have attempted to identify criteria that would allow a decision to be made whether to resect or leave in situ an incidental asymptomatic MD. After a thorough review of the literature, we could not definitively state whether the MD of our reported patient should have been resected or left in situ at the time of the previous LAE. According to our analysis, MD characteristics, patient risk factors and clinical presentation, and the surgical approach (open VS laparoscopic) need to be considered to establish definitive guidelines for the management of incidental asymptomatic MD. Considering the surgical approach, complications that might discourage open surgery and resection are less common with laparoscopic diverticulectomy. In the absence of definitive guidelines, expertise and common sense remain the main resources for the surgeon approaching an incidental asymptomatic MD.

## Figures and Tables

**Table 1:** Studies that do not recommend resection of incidental MD.

Author	Year	Conclusion
Weinstein et al. [19]	1962	Wide-mouthed MD were not considered dangerous, and therefore their resection would only add to the risk of surgical complications and do not provide any benefit.
Soltero and Bill [20]	1976	To save one patient’s life from the complications of MD, it would be necessary to remove approximately 800 asymptomatic MDs. This would be likely to incur a significant increase in postoperative morbidity. They suggest that the prophylactic removal of MD is rarely, if ever, justified.
Leijonmarck et al. [12]	1986	In adults, an incidentally discovered, symptomless MD should be left in place.
Peoples et al. [21]	1995	Incidental diverticulectomy in adults should be abandoned.
Stone et al. [22]	2004	Removal of asymptomatic MD, particularly in women, is not recommended.
Zani et al. [18]	2008	Leaving an incidentally detected MD in situ reduces the risk of postoperative complications without increasing late complications. A large number of MD resections would need to be performed to prevent 1 death from MD. The above evidence does not support the resection of incidentally detected MD.

**Table 2:** Studies that recommend resection of incidental MD whenever found or according to risk factors or other conditions.

Author	Year	Conclusion
Mackey et al. [23]	1983	Recommend resection when risk factors are present: age ≤ 40 years, length of the diverticulum ≥ 2 cm, presence of heterotopic tissue, and male sex.
Vane et al. [24]	1987	Resection of asymptomatic vitelline remnants in early childhood is reasonable at the time of laparotomy for other conditions.
St-Vil et al. [25]	1991	MD discovered incidentally should be resected if ectopic mucosa present or if attached to the umbilicus or to the mesentery by fibrous bands.
DiGiacomo et al. [26]	1993	Stapler diverticulectomy is appropriate and safe and should be done for diverticula that easily fit the device. Diverticula that are so broad-based or short that stapled excision cannot be easily accomplished should be left in situ, since they are at low risk for complications.
Cullen et al. [27]	1994	Recommend prophylactic resection regardless of age (providing no additional condition, such as generalized peritonitis, would make removal hazardous).
Matsagas et al. [28]	1995	Resection of the unexpected MD can be performed safely with a low complication rate, regardless of the patient’s age.
Chiu et at. [29]	2000	The small bowel diverticula, except for MD, do not need to be treated if there are no significant symptoms.
Groebli et al. [30]	2001	The criteria to resect incidental MD: male sex, age < 40, ASA score, the operation being performed, size and position of the MD, palpable mass, exploration for acute right lower quadrant pain showing no other abnormality.
Onen et al. [31]	2003	Resection of the MD is recommended in all children younger than 8 years, including asymptomatic ones, in the absence of absolute contraindications.
Bani-Hani et al. [32]	2004	Resection of incidentally found MD is not associated with increased operative morbidity or mortality.
Park et al. [14]	2005	Recommend removal when risk factors are present: age < 50 years, male sex, diverticulum > 2 cm, and the presence of histologically abnormal tissue.
Ueberrueck et al. [33]	2005	In cases of gangrenous or perforated appendicitis, an incidentally discovered MD should be left in place, whereas in an only mildly inflamed appendix it should be removed.
Dumper et al. [34]	2006	Criteria to resect incidental MD: younger age at presentation, narrow diverticular neck, previous abdominal adhesions or obstructions, and any palpable or visual abnormality of the diverticulum.
Robijn et al. [35]	2006	Recommend resection with a Risk Score ≥ 6. Risk factors of the score: male sex, patients < 45 years, MD >2 cm, and the presence of a fibrous band.
McKay [36]	2007	Recommend prophylactic resection in patients under 50 years of age.
Zulfikaroglu et al. [37]	2008	Recommend prophylactic resection because it is not associated with increased operative morbidity or mortality.
Thirunavukarasu et al. [38]	2011	MD is a high-risk area for cancer in the ileum. With risk that increases with age and high possibility of curative resection with negligible operative mortality, incidental MD is best treated with resection.
Caracappa et al. [39]	2014	Recommend prophylactic resection.
Kilius et al. [40]	2015	Recommend prophylactic resection.
Jadlowiec et al. [41]	2015	Recommend prophylactic resection in patients of all ages.
Gezer et al. [42]	2016	Recommend prophylactic resection regardless of its macroscopic appearance.
Lequet et al. [43]	2017	Recommend resection when risk factors are present: male sex, age ≤ 40 years, diverticulum length > 2 cm, and presence of macroscopically mucosal alteration noted at surgery.
Blouhos et al. [44]	2018	Recommend resection when risk factors are present: age < 50 years, male sex, diverticulum length > 2 cm, and ectopic or abnormal features within a diverticulum. Diverticulectomy should be performed for long MD and wedge resection for short MD.
Chen et al. [11]	2018	Heterotopic tissue is the main cause of a complicated diverticulum, and it is safe and feasible to remove the incidentally found MD.
Hansen et al. [1]	2018	Incidental MD should always be resected in the pediatric population and in the presence of risk factors in the adult population.
Mora-Guzmán et al. [45]	2018	Recommend prophylactic resection because the benefits of resection of this high-risk area for cancer outweigh the risks of surgery.
Mora-Guzmán et al. [46]	2018	Recommend prophylactic resection of incidentally found MD because of benefits outweighing the risks in this high-risk area for cancer.
Demirel et al. [47]	2019	Recommend prophylactic resection due to the risk of ectopic tissue that may cause a mass effect or a narrow neck that may predispose to obstruction and diverticulitis.
Chen et al. [48]	2021	Recommend prophylactic resection when MD is longer, with a fibrous band at the tip of the diverticulum and a narrow base.
Tree et al. [49]	2023	Recommend laparoscopic prophylactic resection.

## Data Availability

Patients’ data registry of Ospedale di Circolo e Fondazione Macchi (VA).
